# The intersection of finTech adoption, HR competency potential, service innovation, and firm growth in the banking sectors using Entropy and TOPSIS

**DOI:** 10.1371/journal.pone.0313210

**Published:** 2025-01-14

**Authors:** Habib Ullah Khan, Muhammad Abbas, Shah Nazir, Faheem Khan, Yeon-kug Moon

**Affiliations:** 1 Department of Accounting & Information Systems at the College of Business and Economics, Qatar University, Qatar; 2 Faculty of Computer Science and Engineering, Ghulam Ishaq Khan Institute of Engineering Sciences and Technology, Pakistan; 3 Department of Computer Science, University of Swabi, Pakistan; 4 Department of Computer Engineering, Gachon University, Seongnam-si, South Korea; 5 Department of Artificial Intelligence and Data Science, Sejong University, South Korea; Industrial University of Ho Chi Minh City, VIET NAM

## Abstract

The adoption of Financial Technology (FinTech), along with the enhancement of Human Resource (HR) competencies, service innovation, and firm growth, plays a crucial role in the development of the banking sector. Despite their importance, obtaining reliable results is often challenging due to the complex, high-dimensional correlations among various features that affect the industry. To address this issue, this research introduces a hybrid Multi-Criteria Decision-Making (MCDM) model that integrates the Entropy-Weighted Method (EWM) and the Technique for Order Preference by Similarity to Ideal Solution (TOPSIS). The primary objective of this study is to systematically evaluate and rank multiple alternatives based on key criteria using the EWM-TOPSIS approaches. Specifically, the analysis considers eleven multifaceted characteristics and eight potential alternatives (A_1_ to A_8_), revealing the significant influence of the proposed MCDM approaches in assessing FinTech adoption, HR competency, service innovation, and firm growth. The findings underscore the effectiveness of the entropy-TOPSIS approaches in providing a structured analysis for a smarter and well-informed decision-making. Ultimately, this research proposes the best alternative from the evaluated options, contributing valuable insights into the future role of FinTech, HR competencies, service innovation, and firm growth within the banking sector.

## Introduction

In today’s evolving technological context, numerous industry activities must go through digitalization. By using modern technological systems (e-banking), service innovation (e-branch shops), and employees, banking institutions may introduce alternative service mediums while simultaneously lowering operational expenses by decreasing the requirement for branch offices and people. Human and banking sector reliance on technology has evolved noticeably as a consequence of the most obvious advances in computer innovation, which has been found by studying different articles. As shareholders and corporate officers predict that banking markets will employ innovation to solve shortcomings in the post-crisis scenario and upend conventional wisdom, the banking industry will surely experience this changing experience. This category of innovation has been given the moniker "finance technology," or, more frequently, "FinTech." Fintech innovations and HR competency potential have gained importance in the banking industry since they offer more sophisticated techniques to engage with consumers and gather tangible data to help with lending decisions [1] and solve customer-related issues efficiently.

The banking sector’s continuous digitalization is crucial for enabling institutions to enter a data economy populated by tech-savvy consumers. Traditional and cutting-edge banking services are being provided by technologies and innovations, making the shift from the "business as normal" philosophy easier. This shift is facilitated by the use of technologies and innovations, ensuring the success of the banking sector. The adoption of FinTech and service innovations in the banking sector [2] focuses on various topics such as theft, anti-money laundering, cyberattacks, performance outcomes, advertising strategies, and customized services. The sector is also aiming to lower operational costs, balance rapidity and adaptability, and adopt new approaches like FinTech, HR capabilities, and service improvement. The traditional banking paradigm is being replaced with a technologically modified one, requiring advancements in database administration and intelligent wealth creation. Researchers face challenges in evaluating FinTech adoption, HR competency potential, service innovation, and firm growth’s impact on the banking sector. This study introduced a well-known hybrid MCDM model for the efficient evaluation of these innovations and their adoption in the banking sector. The hybrid model includes an integrated EWM and TOPSIS to solve the multi-criteria issue and arrange the alternatives sequentially for effective decision-making in the banking industry. The EWM technique is applied for the measurement of criterion importance while the TOPSIS system is applied for the ranking of chosen choices (alternatives). The best choice is identified among several options and an efficient decision is made.

The research [3] have explored the impact of some of the most tremendous scientific resources used by banks on their marketing strategy, with a concentration on the advancement of human assets and conventional advantage ratio in the field. Knowing this in advance, the proposed article will examine how digitalization has affected the efficacy and strength of social resources in the European banking system in hopes of addressing the shortage in the literature on intangible resources that this latter aspect represents. The analysis for the proposed survey indicates how financial institutions are shifting away from a more wealthy business plan and away from millennia of corporate practices. It also demonstrates how a surge in the skill level and quantity of exceptionally competent human assets is forming the latest concept. The study [4] have offered a succinct summary of the evolving business and FinTech attitudes that banks have developed as a reaction to rivalry from the fintech sector. It emphasizes the crucial principle that one must take into account the connections between FinTech and restructurings and organizational values in order to appropriately assess the FinTech breakthrough trend. Against the backdrop of FinTech innovation, business structure and internal architecture for banks have received little interest among scholars up to now. Yet, by developing a structure through which these linkages may be comprehended, the proposed study demonstrates their significance and value.

Intellectuals, corporations, and financiers are becoming more interested in the "FinTech" paradigm, which combines the banking industry with digital technologies. Fintech is frequently covered in the media, which affects public perception and raises knowledge, but real proof must back up this comprehension. In order to evaluate the existence of FinTech advancements, the proposed study might investigate five Swiss FinTech companies within the theoretical structure of existing FinTech knowledge and its characteristics. As a result, the analysis broadens the comprehension of fintech and creates a favorable environment for further investigation. The primary objective of the proposed research [5] is to ascertain if FinTech will revolutionize the monetary system in a way that enhances the health and viability of the banking sector. In hopes of understanding the forces driving fintech and its possible implications, the research advised concentrating on the European financial sector. The analysis offers several helpful guidelines for legislators and European financial institutions intending to boost their standing in modern finance and keep an eye on the potential complications. The paradigm for customer service has long since changed as a result of digitization in the banking sector. Experts in the fields of computer management and banking are studying the impact of technological progress on fintech organizations. Concerning these cutting-edge and distinctive corporate activities [6] have done extensive studies on the most current advancements in digital banking analysis. The Financial Technology Cube offers a methodology for examining this area and comprises the main components of fintech and financial innovation, including the entities involved, the technology and technical theories employed, and the pertinent business processes. This method makes it possible for scientific research to be organized in relation to one another, encourages the recognition of potential research topics, and helps those in academia working in financial development to find their way.

The existing work concentrates on the incorporation of financial technologies in traditional banking systems; nevertheless, it is unable to present a thorough assessment system that combines cutting-edge technologies like blockchain and artificial intelligence with tried-and-true growth factors like customer service and regulatory compliance. Furthermore, few studies are using MCDM approaches to investigate mechanisms for dynamic criteria weighting in conjunction with sustainable practices. The purpose of this research is to propose an adaptable MCDM approach to assessing the various alternatives by considering multifaceted criteria and also evaluate how FinTech adoption in the banking industry interrelates with service innovation, HR competence enhancement, and organizational growth. The following are the main goals of this research article;

To review the adoption of FinTech, service innovation, HR competency potential, and firm growth in the banking sector.

- To propose an effective multifaceted approach for the intersection and selection of a better alternatives affecting the banking sector’s performance and development.
- To rank various alternatives operating within the banking sector by evaluating and selecting the most effective characteristics derived from the analysis.
- To utilize a hybrid Multi-Criteria Decision-Making (MCDM) system to systematically assess and rank different alternatives, employing the Entropy-Weighted Method (EWM) for criterion weight determination and the Technique for Order Preference by Similarity to Ideal Solution (TOPSIS) for alternative ranking.
- Finally, to identify the most effective alternative that significantly contributes to enhancing the environmental efficiency and overall growth of the banking sector.

The article is divided into the following sections for the remaining portions. The next section looks at existing works of literature. The study methodology and extensive computations are presented in the third section. The Fourth section contains the results as well as an explanation of the research methods. Our debate comes to a close in the Fifth section, where we also summarise the results and suggest future study areas.

## Literature review

The rapid advancement of computer technology has accelerated innovation in the banking sector, attracting academic interest and fostering the formation of numerous commencement businesses due to the use of advanced innovation and flexible entrepreneurial processes. These businesses are swiftly being recognized as competitors for existing organizations, particularly banks. The work [7] reveals the analysis of Indonesia’s growing Fintech financing industry as a subset of business model innovation. Millennials, smart people who are known as competitive and quick executives rule the Fintech loan sector. Digitalized innovations, which are constantly developed to satisfy the evolving needs of consumers, help to enhance their strategic benefits. Besides the increasing needs it confronts, Fintech lending’s explosive development is crucial for expanding a person’s ability to obtain financing. The paper [8] analyzes the relationship between HRM practices and the intention to remain in the organization, as well as the intermediary effects of two variables: purpose similarity and a cognitive approach to HRM. Applying a sequential framework to a sample of 265 accredited auditors working for CPA organizations, we reveal that expertise and organizational dedication moderate the influence of mobility and recognition. Furthermore, the interaction between information-sharing, fair remuneration, and the decision to remain is mediated by organizational commitment. Training significantly impacts stay desire. These outcomes pave the way for the creation of an HRM paradigm that links expert personnel’ organizational and specialist devotion, as well as the prospect of reaching a compromise between traditional ethical behavior and organizational success.

All nations, like India, that wish to experience wealth creation must pay particular attention to their banking industries. In order to secure India’s long-term prosperity [9] have aimed to ascertain the present achievement criteria for bankers and argue that regulators must raise their satisfaction ratings. Data on demographics has been assessed using the SPSS tool. In addition, features that bankers who employ the SmartPLS software require to remain satisfied at work were revealed using multivariate techniques. The results of research [9] reveal that elements like pay, rewards, and performance reviews have a big impact on how satisfied Bangladeshi bank employees are with their careers. The suggested work’s premise is that corporate and covered entities ought to ensure that assessments are performed and that those who conduct them ought to be paid remuneration for their work. It investigated how knowledge proactiveness desire, a creative, unique, and finite resource, effects arise firm performance using the foundation of the Resource-based paradigm. The paper [10] premise states that the environment for creativity has an impact on innovativeness, which in turn has an impact on practical corporate strategy, innovation strategies, as well as a person’s demand for knowledge responsiveness and inventive thinking styles. Three time periods were used to acquire a statistically isolated data set from personnel of Pakistan’s financial industry in Islamabad. The results of this analysis, which were centered on the PROCESS method, suggest that intellectual capital regulates the link between the incentive for knowledge responsiveness and functional business success. The atmosphere for creation was found to have an impact on the relationship between innovativeness and functional firm efficiency, and the creative cognitive model was shown to facilitate the connection between knowledge proactiveness desire and technology.

The proposed study examines the association between bank revenue and FinTech deployment by utilizing a panel fixed-effect regression framework to investigate a sizable set of European banking systems. The findings of the study [11] indicate a beneficial association between financial innovation and green financing, which may be ascribed to the successful creation, analysis, and tracking of technological advances. In addition, the findings demonstrate a clear correlation between FinTech expenditure and the uncertain return on capital related to minimum budget, a wider range of product offers, and reduced economic resources. Furthermore, studies indicate the significance of factors like company size, trading volume, and wealth creation effectiveness in impacting banking performance and green financing choices. The consequences of these findings are crucial for comprehending how FinTech integrates into sustainable financial and environmental objectives. Digitalization is driven by several factors, such as the need to stand out from rivals, provide customer care in sites without branch offices, and cut expenditures. The case’s use of modern technology raises several questions. Hence, the extent to which the Greek banking industry has embraced technological change was analyzed. The elements of the Technological Acceptance Model were investigated using a multivariate regression technique. The findings of the research [12] reveal how bankers feel about modern technology. Greek financial institution administrators have been provided with a helpful instrument to create special training initiatives that will aid staff members in adapting to the evolving electronic medium. If workers are willing to embrace and incorporate technology into their everyday work processes, it would be helpful for administrators to recognize it.

The study [13] demonstrated how big data as a tool and technology has distinguished its status as a formidable and cutting-edge tool that helps the Indian financial system both detect security dangers and corrupt practices as well as effectively prevent them from occurring. Empirical quantitative research findings through Systematic Literature Review [14–17] utilizing the Vos Viewer software and Web of Science libraries show that big data technology is a critical strategy to implement in the Indian financial sector. This would improve academics’ expertise and abilities in this domain by assisting them in better understanding the various big data tools and strategies utilized for reducing banking investment issues and their purpose in functional banking process monitoring. The paper [18] has examined the profitability of major banks and how it influenced Effectiveness by putting the economic power of effective design theory into practice. The Herfindahl Hirschman Index (HHI) and sales growth were evaluated as a stand-in for an effective architecture in a survey that contrasted Singapore and Pakistan from 2005 to 2020. It has been found that Singapore has a monopoly market situation and a supersaturated industry. Pakistani financial sector, on the other hand, suggests a completely challenging market. Verify the co-integration and long-term connections between the components by using the vector error correction (VECM) methodology.

The aim was to determine how the bottom lines of banks are impacted by automated teller machines (ATMs), the web, and wireless technology [19]. The research work [20] looks at whether fintech causes firms to release more or fewer loans. The analytical results show that using digital wallets decreases SME loans, which means that basing credit choices on real facts lessens the possibility that SMEs would utilize bank loans. The results are in favor of strengthening intimate relations between financial institutions and business owners.

The corporate sector has experienced a challenging transition in the past decade, necessitating businesses to rapidly develop new products and services through innovative IT technology or creative business models, ensuring they maintain an attractive lead [21]. It emphasizes the need for internal integration at the core of a business’s activities and establishes a linkage between IT development and sustained corporate success. Based on the findings of major organizations and SMEs in the financial and telecom areas, an explorative technique was used to develop broad conclusions. The paper [22] assessed financial market trends, both inside and outside variables that influence financial institutions ability to compete, and the function and impact of digitalization in providing banks with strategic strategies like reduced circulation expenses, higher labor efficiency, risk-adjusted returns, and effective management determinations. Data on how technology has impacted banks’ capacity to compete and efficiency ratings are provided. The causes for modernization in the financial industry are examined in addition to examining the issues and remedies found.

The results of the research [23] improved human comprehension of work engagement by studying the role of consciousness as a mediator in the link between observed entrepreneurial mindset and workplace ethics. The analysis of the information gathered from a poll of 404 commercial bank workers using structural equation modeling. The magnitude of the investigation is categorically supported by the findings of the confirmatory factor evaluation and internal consistency. The findings of a reserve bank quantitative study research show that individuals’ opinions of significant degrees of entrepreneurial attitude have a beneficial influence on their willingness to take risks in the workplace. The analysis contains suggestions for financial industry experts on how to encourage imaginative employment practices via self-leadership and an innovative approach. FinTech is the integration of technological advancement and creativity to provide investors with banking goods and services. The paper [24] aimed to analyze how fintech is affecting the efficiency and competitiveness of the financial sector in the United Arab Emirates. 76 bankers and analysts from Dubai are used in an experimental evaluation of the study. The analysis shows that the United Arab Emirates’ financial firm’s efficiency and comparative outputs have been significantly impacted by the use of technology. The second conclusion contends that the success of the financial sector in the United Arab Emirates is directly influenced by Financial technology acceptance and innovation administration synchronization. The suggested research is necessary because of the nearly two hundred different ethnicities employed in the UAE financial system as well as the importance of FinTech and its competitive nature to the sector’s growth.

As banks typically have a connection to the entire monetary sector and the power to make legal choices, the financial industry has been recognized as the main route for hiding illegal revenues. Banking firms and their procedures are easily accessible to fraudsters and individuals who sponsor violence. The article [25] have concentrated on conformity and anti-money-laundering regulations in the financial industry utilizing recently innovative technology namely blockchain. The analysis also discusses AML rules and pays particular attention to concerns with the fabrication of KYC and the financial strain on firms. The analysis further offers findings and suggestions for advancing cutting-edge blockchain technology while tackling issues with ML. It also incorporates blockchain technology’s ability to give financial organizations the most recent regulations and guidelines. Information relating to how the financial services market affects creative output varies amongst industrialized nations. China has a distinctive banking industry that is governed by the state. There is still a paucity of awareness among researchers as to how varied financial systems in underdeveloped nations affect creativity. The research [26] have used Chinese prefecture-level towns to study how a local invention was influenced by bank profitability to close this development gap. The suggested research concluded that local development was greatly affected by financial services rivalry. After robustness testing, the conclusion is still valid, and it is particularly evident for areas of eastern China and areas with extensive safeguards for intellectual property. Potential strategy, service simulation techniques, and breakthroughs all benefit from financial rivalry. Moreover, the data demonstrates how bank rivalry encourages local development by expanding the lending capacity. The state may think about enhancing safeguards for intellectual property and privatizing bank management in order to promote local entrepreneurship.

To analyze the various methods that monetary fluctuations affect R&D investment and copyright action [27] created a horizontal R&D economic strategy. Initially, a "micro" change that eliminates obstacles in the financial industry reduces borrowing costs, which increases productivity and budget considerations in R&D. The second adjustment is "macro" in nature and loosens regulations on financial assets and credit criteria. Whereas this change boosts flexibility, it also raises the possibility of bankruptcy, which might lead to increased lending rates. Hence, the suggested framework contends that although micro changes promote creativity, macro changes might have the opposite effect. The study used real facts to back up these conclusions across a selection of 21 OECD nations. The paper [28] look into the connection between leading financial growth and the rise of the industrial sector using divisional statistics from India. The results emphasize the role of financial service expansion in the growth of the town’s industrial sector. The suggested study emphasizes the value of region-financing innovation by proving that region financing, as opposed to district-wide financing, positively affects domestic manufacturing growth. The study also shows that access to industrial loans is less restricted in areas with greater literacy rates.

### Use of MCDM methods in the assessment of countries/regions in the development of digital technologies in enterprises, including the banking sector

The research explores the crucial role that technology innovation and digitalization have in shaping the economies of different nations, particularly within the system of the European Union (EU) [29]. Various factors are necessary to build a competitive, knowledge-driven economic structure. The creative and digital projects businesses embark on to promote economic growth are highly valued by the European Union. It also acknowledges the vital significance of a sustainable future. To ascertain the frequency of these two variables in each of the 27 EU member states, a thorough investigation was carried out. The objective was to investigate the relationships between technological advances and digitalization, as well as the effects of economic, social, environmental, and energy-related characteristics. The investigation adopted a new approach that integrates several MCDM frameworks (EDAS, MOORA, and VIKOR) with weighting procedures (CRITIC, Entropy, Laplace criterion). The results of the research [30] reveal that investment in technological advancement, capability for innovation, and digital transformation are positively correlated. Moreover, the "old EU-14" members have advanced more quickly than the "new EU-13" countries.

Small and Medium-Sized Enterprises (SMEs) as discussed in research [31] need to go via a mechanism of digital transformation prior to the efficient integration of digital initiatives within a specific nation or region. The EU recognizes the imperative for SMEs to rapidly embrace digitization in order to establish a knowledge-based economy that is both competitive and sustainable, which has prompted substantial efforts to expedite this transformation. The primary objectives of the study [32] were to assess the degree of digital maturity among SMEs within the EU-27 and to investigate the influence of national economies on this developmental trajectory. The investigation employed data sourced from the Eurostat database and incorporated eleven Industry 4.0 indicators, utilizing Principal Component Analysis (PCA) to elucidate national commonalities. Furthermore, the entropy-based EDAS methodology was applied in each country to evaluate the digital maturity of SMEs. The statistical tests of Spearman and Kendall Tau were utilized in offering a deeper analysis of the causal connection between digital development and other economic factors. The analysis indicated notable differences in the digitalization efforts of SMEs between the more recent and more established member states of the European Union. This underscores the necessity for focused initiatives designed to accelerate digitalization, especially in the countries that are presently trailing behind.

SMEs have a significant challenge of retaining their competitive edge in the field of technological change. But sometimes their expansion is hampered by a lack of resources. To address this, several countries offer environments of assistance to help SMEs become digitally mature. The techniques that are now in practice are often theoretical or created with larger firms in mind, even though evaluating digital maturity is essential to give pertinent advice. A multi-attribute model for assessing SME technological maturity was created in response, using a design science research methodology [33]. The model leverages the more sophisticated multi-attribute Decision Expert (DEX) technique, which has been tried and tested on real-world scenarios and evaluated by experts in the area. The approach worked well in real-world business settings, providing practical viewpoints to analyze and guide the technological changes of small and medium-sized enterprises.

It is concluded that the MCDM techniques are crucial for assessing the ways in which digital technologies are impacting enterprises worldwide, particularly those in the banking industry. These methodologies offer a thorough assessment by considering an extensive range of attributes that demonstrate the advancement of innovation and digitalization within firms. The utilization of MCDM methodologies further facilitates more intricate cross-national comparisons, as these techniques integrate a set of multifaceted factors, including economic performance, investment in research and development, and technological adoption. This is particularly salient in the banking industry, where the imperative of digital transformation necessitates that MCDM methodologies effectively navigate the trade-offs between the merits and demerits of digital infrastructure and innovation capacity. The obtained outcomes can be leveraged to inform strategic planning and policymaking initiatives aimed at fostering technological innovation and enhancing competitiveness.

### Problem statement

The existing literature mainly focuses on the adoption of financial technologies in traditional banking, but there is a lack of a comprehensive evaluation framework that integrates emerging technologies like blockchain, artificial intelligence, and digital banking with conventional growth factors like customer service, regulatory adherence, and financial efficacy. The significance of sustainable practices in banking and green supply chains is also under-researched, with a scarcity of studies using MCDM methodologies. The research lacks an exploration of dynamic and adaptive criteria weighting mechanisms that can adapt to market dynamics, consumer preferences, and technological advancements. Holistic performance metrics are needed to encompass not only financial aspects but also technological, environmental, and social dimensions of banking sector expansion, as existing frameworks tend to prioritize financial metrics. This study aims to create an adaptive MCDM framework for evaluating banking sector growth, considering factors like FinTech adoption, HR competency potential, service innovation, and firm growth. The framework uses Entropy-TOPSIS for dynamic weighting and ranking criteria, ensuring adaptability to changing industry conditions. The goal of this study to create a comprehensive decision-making instrument that helps banking organizations balance financial expansion with technology and sustainability efforts, addressing existing performance evaluation deficiencies. This research highlights the need for a flexible decision-making strategy in the banking sector, leveraging MCDM methodologies to consider a wider range of growth determinants.

## Methodology

The banking sector has been facing constant customer service evolution due to digitalization for years. Its development is highlighted by more interconnection and quicker information interpretation, across the user interface and in back-office tasks. Now, the focus of technology has switched from enhancing the performance of standardized tasks to offering fundamentally novel possibilities and paradigms for suppliers of the banking system. Besides this improvement, HR competency potential, company expansion, and service innovation might influence the growth of the banking industry, bring about various changes to classic banking mechanisms, and modernize them by utilizing cutting-edge technology and procedures. Various MCDM techniques [34] are utilized to solve multi-criteria optimization problems. Appropriate features or characteristics are selected from a large group of characteristics or features [35]. We suggested Entropy-based TOPSIS algorithms to assess their potential contribution to the banking sector. These methods effectively rate the alternatives and evaluate their relative relevance for a better choice. As the alternatives are ordered using TOPSIS, Entropy is used to determine the criteria relevance. In this study, a good and influential alternative is identified.

**Fig 1:**
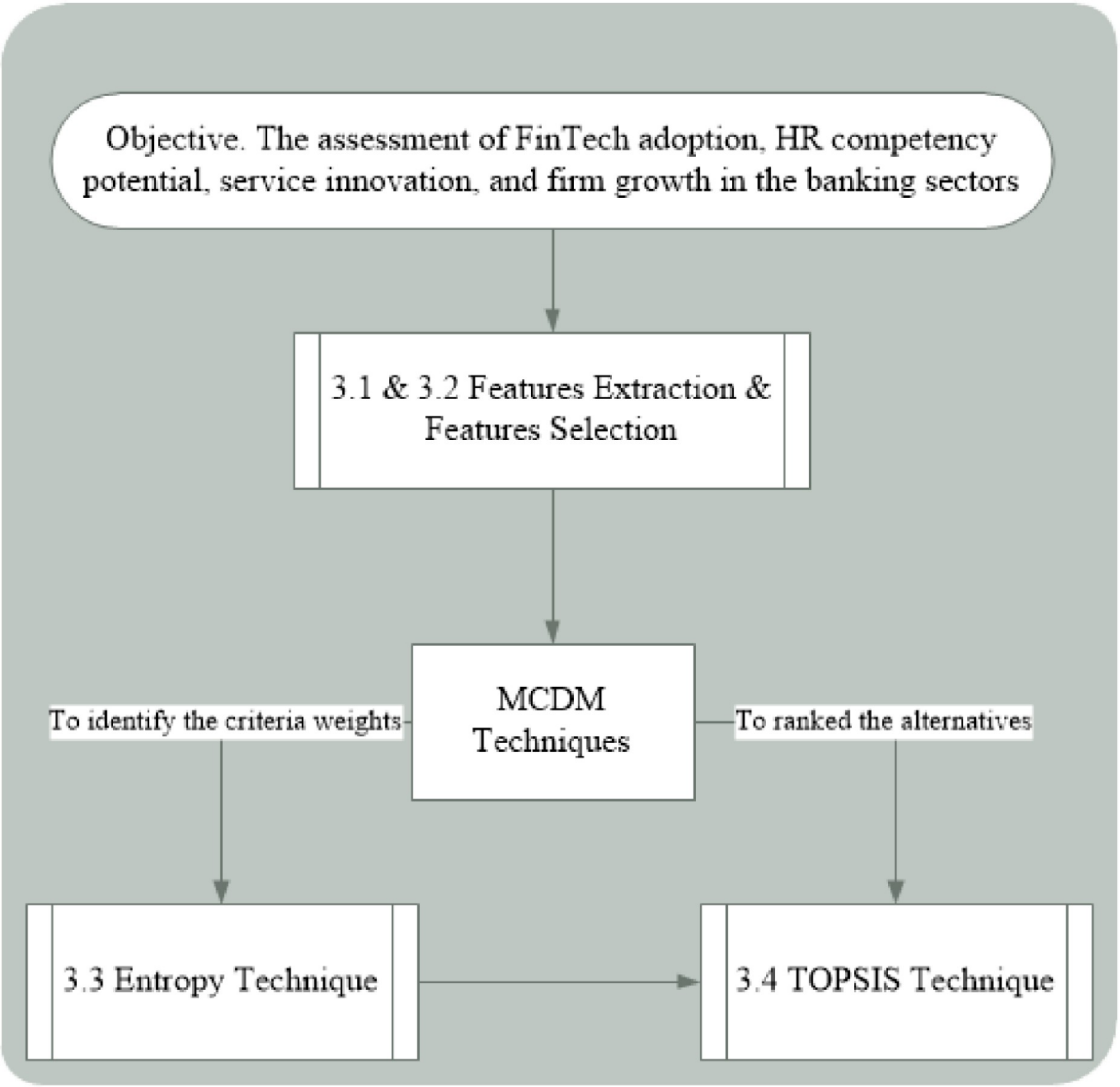
Research methodology.

The assessment of different things and technologies including FinTech adoption, HR competency potential, service innovation, and firm growth, and their significant role in the banking sector are crucial for stakeholders and bankers. For this purpose, we apply two MCDM-based Entropy and TOPSIS techniques as discussed in the studies [36, 37] to measure the criterion importance and rank the alternatives. The assessment of these technologies and their role in the banking sector are efficiently completed effectively. The assessment of a set of different alternatives is done and an effective and appropriate one is selected that has played a significant role in the development of the banking sector. The objective of this research is to analyze FinTech adoption, HR competency potential, service innovation, and firm growth and their significant role in the banking sector. After defining the goal, we extract common and significant features from the literature and select a few suitable ones from them. These identified and selected criteria are assessed and their weights are determined using Entropy, although the alternatives are ranked using TOPSIS. The significant role of these technologies is examined and the best alternative is selected in this mechanism. Fig 1 depicts the hierarchical structure of this analytical methodology.

### Features extraction

Features extraction plays a vital role in the evaluation and decision-making procedure. In addition to this, the most significant features are taken from a wide range of articles that are examined and found to be pertinent to this field. Table 1 displays the extracted characteristics.

**Table 1:** Extracted features.

Authors and Citation No.	Features
Akyuwen et al. [7]	Information technology, intellectual curiosity, adaptive, Fintech lending, agility, aggressive, competitive, digital technologies, substantial, dynamic
Brodny and Tutak [29, 30]	Capital markets, credibility, conformity, contingents, predispositions, convictions, retaining, digital technologies, independence level
Gupta and Verma [9]	Economic growth, job pleasure, satisfaction level, stability, demographic information, performance assessment, early prevention, bank profitability
Naseer et al. [10]	Assumptions, inimitable resources, proactiveness motivation, operational, firm performance, open innovation, interactive, cognitive style, contextual resources
Mirza et al. [11]	Technological innovations, financial services, market concentration, bank profitability, green lending, efficiency monitoring, workflow management, risk-adjusted, capital efficiency
Kitsios et al. [12]	Digital transformation, e-banking, operating cost, acceptance rate, perception, facilitation, digitalization, e-services, user’s intention, connectivity
Singh et al. [13]	Money markets, paperless transactions, digital transformation, risk monitoring, innovative instruments, fraudulent behavior, early prevention, workflow management, financial risks
Talpur [18]	Competitiveness, performance, market power, efficient structure, comparative, concentration ratio, competitive environment, co-integration, bank profitability
Fasano and Cappa [20]	Financial innovation, benefits, conceptualization, technological adoption, e-banking, innovation diffusion, data availability, bank performance
Marhraoui and El Manouar [21]	Fintech services, effective, interactive, codifiable data, decisions, human interactions, soft information, e-banking, information asymmetries
Cao et al. [34]	Business environment, turbulent, competitive advantage, IT innovation, sustainability, bank performance, economic impacts
Gabbasova et al. [22]	Banking competition, competitiveness, digital technologies, competitive advantages, circulation costs, labor productivity, risk optimization, managerial decisions
Kör [18]	Innovative, working behavior, performance, self-leadership, entrepreneurial orientation, reliability measures, confirmatory factor, economic activity
Dwivedi et al. [24]	Fintech innovation, financial services, competitiveness, performance, significance, cash management, digital currency, digital factoring
Thommandru and Chakka [25]	Accessibility, legal authority, banking mechanism, fund transfers, expensiveness, compliance, emerging technologies, financial services
Huang et al. [26]	Banking competition, innovation activities, regional innovation, robustness, design innovation, credit supply
Boikos et al. [27]	Financial reforms, patent activity, credit controls, bank reserves, borrowing costs, default rates, reserves paradox, deposit rates, innovation
Thampy and Tiwary [28]	Financial development, manufacturing growth, lending technology, bank credit, credit availability, literacy level, human capital, economic activity

#### Features selection

**Fig 2:**
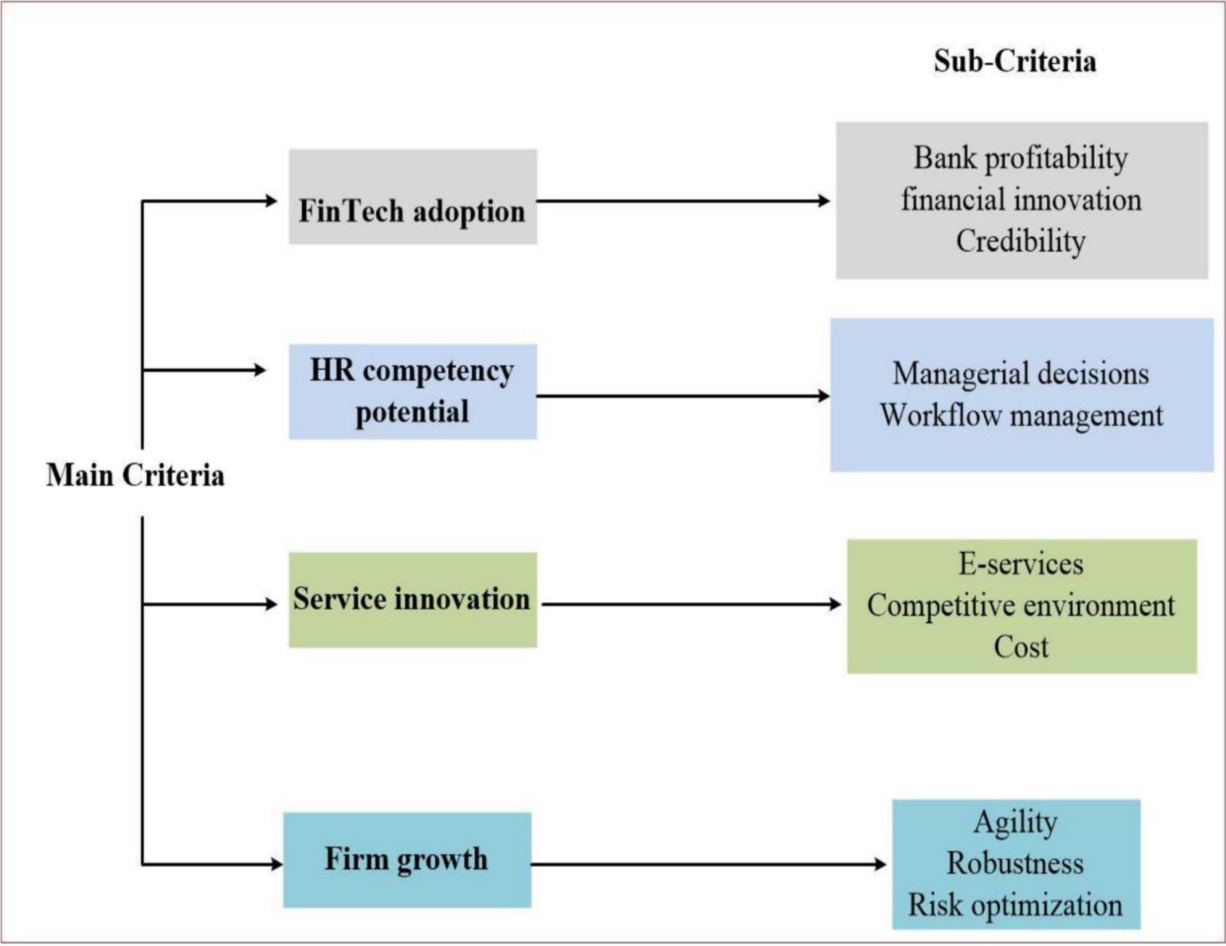
Selected criteria and their subcriteria.

The most significant and useful multifaceted attributes are chosen in this research based on their higher relevance and performance in the banking sector. The selected criteria and their subcriteria are as listed in Fig 2.

**Fig 3:**
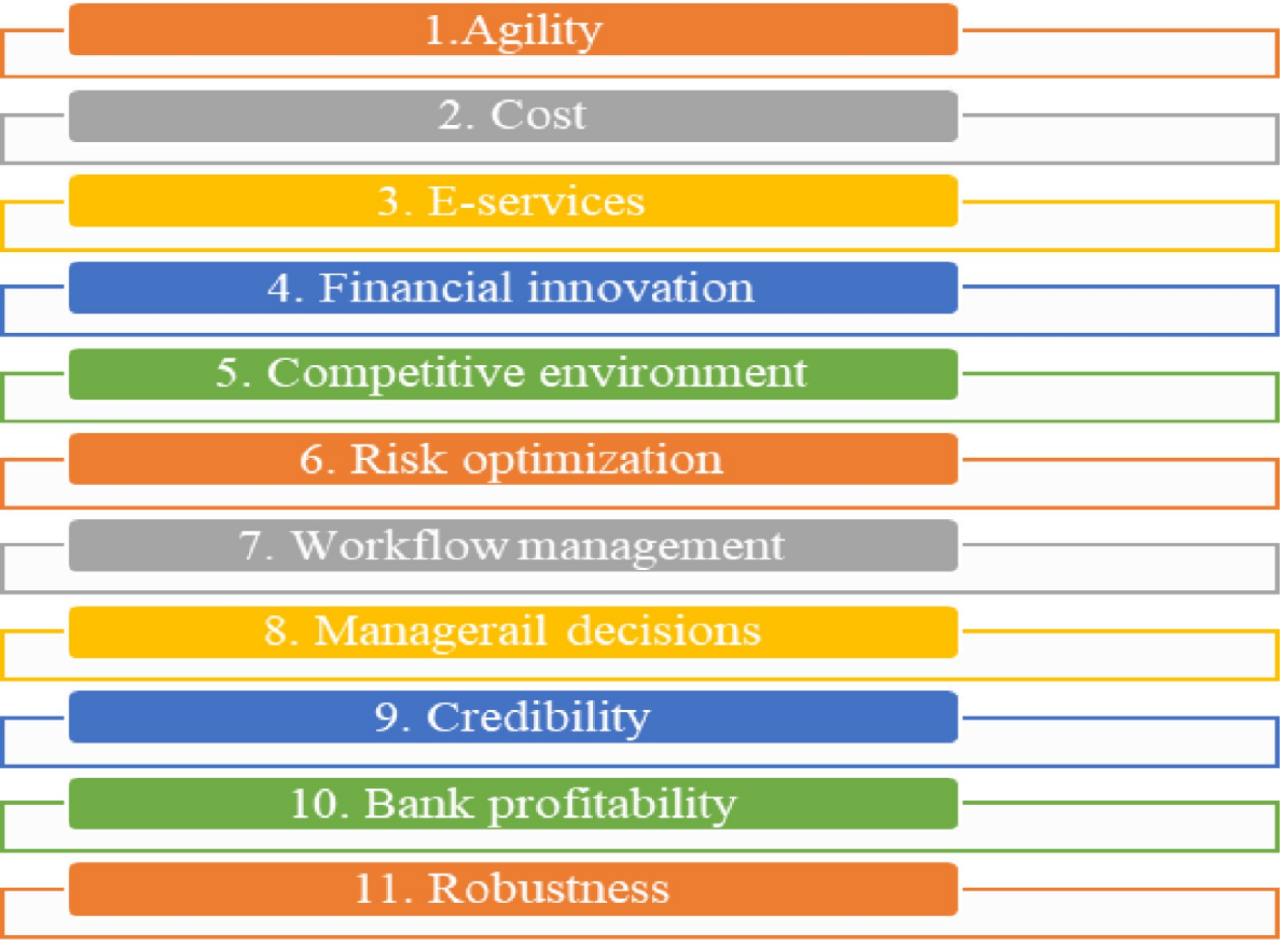
Selected criteria.

The list of the sub-characteristics determined and selected for evaluation is as depicted in Fig 3.

### MCDM model

The proposed hybrid Multi-Criteria Decision-Making (MCDM) model, incorporating Entropy and TOPSIS approaches, is instrumental in the banking sector for the evaluation and prioritization of various alternatives based on the certain criteria. The utilization of the entropy approach helps in the determination and measurement of the relative importance of diverse factors like FinTech adoption, HR competency, service innovation, and firm growth, enabling decision-makers to assign appropriate weights to each criterion. Subsequently, the TOPSIS approach facilitates the ranking of alternatives by identifying the best options that are closest to the ideal solution while being farthest from the worst. This integrated model facilitates banks in making well-informed decisions, enhancing resource allocation, and improving overall performance in a rapidly evolving financial landscape.

### Entropy weighted method

**Fig 4:**
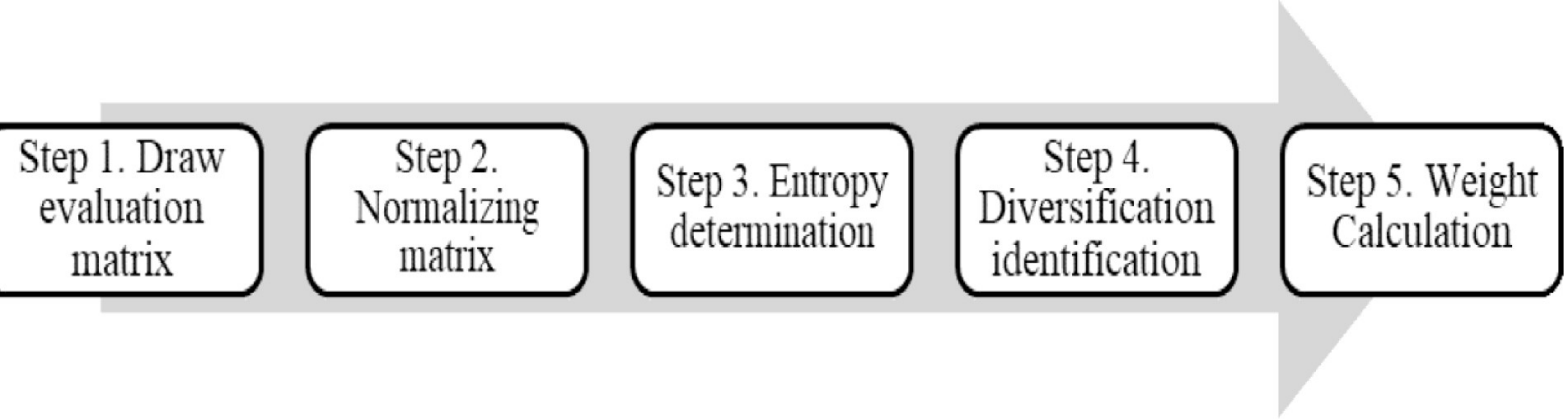
Steps involved in Entropy.

The entropy approach is a renowned multifaceted statistical technique utilized to ascertain the relative importance of different factors. Assessing the data each criterion provides helps decision-makers understand how various factors compare in significance. Normalization of the decision matrix is the initial stage toward ensuring the impartiality and fairness of the analysis. After the data have been normalized, the entropy ratio for every criterion needs to be calculated. This value displays the degree of variability or uncertainty in the data; a lower entropy means that a particular criterion influences the decision-making process more significantly. The determined entropy values are the source of the weights allocated to the criterion. Higher weights are assigned to criteria with lower entropy, indicating their significance to the assessment as a whole. The entropy method has been a useful tool for the banking industry for the determination of what factors—like operational effectiveness, incorporating new technologies, or service quality—are most important for fostering business expansion or enhancing HR capacities. Banks may make smarter and strategic decisions that are in line with their goals by putting a numerical value on these factors. The steps involved in this approach are as described in Fig 4.

#### Numerical work of Entropy

The Entropy technique is implemented in this methodology for the efficient determination of criterion importance and analysis of FinTech adoption, HR competency potential, service innovation, and firm growth in the banking sectors. The most effective and comparable characteristics are extracted and chosen for the evaluation mechanism. A set of four main criteria are selected consisting of Fintech adoption, HR competency potential, Service innovation, and firm growth that have further divided into eleven subcharacteristics including agility, cost, e-services, financial innovation, competitive environment, risk optimization, workflow management, managerial decisions, credibility, bank profitability, and robustness while eight different banks are taken as alternatives in the current scenario. These all subcriteria without cost are beneficial. To weight and compare the chosen subcriteria, the entropy mechanism is utilized. The entire calculation of this process is as follows;

*Step 1*. *Decision matrix*. The goal of assigning a numerical value to each attribute on a grading scale that ranges from one to nine is to generate a matrix that accurately demonstrates the expert panel’s opinions. This statistical scale allows for a more thorough review of the effectiveness of every option and improves the entire evaluation’s correctness. The lowest efficiency or performance is represented by a rating of one, which denotes slight usefulness or appropriateness for one criterion over another. On the other hand, a score of nine denotes the highest level of accomplishment and indicates exceptional effectiveness in line with the expected results. This thorough grading system enhances the evaluation’s ability to determine even the smallest differences between options, increasing the accuracy of the findings. This decision matrix containing eight alternatives and eleven characteristics is outlined in Table 2 and serves as the foundation for the existing framework for assessment and decision-making. This systematic mechanism facilitates improved comparative calculations as well as informed decision-making about FinTech integration, potential for HR capabilities, service innovation, and organizational advancement in the banking sector. This offers an in-depth mathematical assessment in which each choice compares to the specified criteria, ensuring the results obtained are reliable and appropriate.

**Table 2:** Decision matrix.

Criteria Alternatives	Agility	Cost	E-services	Financial innovation	Competitive environment	Risk optimization	Workflow management	Managerial decisions	Credibility	Bank Profitability	Robustness
A_1_	2.60	5.50	3	7	8.9	4	6.85	9	2	5	3.5
A_2_	5	3	8.5	2	5	9.2	4.15	7	4	6.35	8
A_3_	8	6.75	5	4	9.3	3	5	2.5	7	2	6.9
A_4_	3.75	8	4	8.5	2	6	7.4	5	3	8.65	4
A_5_	6	2.5	7	3	6.7	2	8	4.4	9	4	7.9
A_6_	7.5	4	5.9	5	7	8.8	2	6.75	5	3	9.6
A_7_	4	7.25	8	2.5	4	5	3.6	3	8.85	5.7	2
A_8_	9	5	2.6	6	7.1	7	3	8.35	6	4.3	5.1
$\sum_{j=1}^{n}a_{\mathrm{ij}}$	45.85	42.00	44.00	38.00	50.00	45.00	40.00	46.00	44.85	39.00	47.00

*Step 2*. *Normalized matrix*. Use Algorithm (1) to produce the required normalization matrices, as seen in Table 3. All parameters and alternatives were handed a tentative weight before the testing. Alternatives varying from A1 to A8 are available. The normalization process has been performed to reduce the subjectivity and remove errors. Comparisons between the various choices based on their criteria are explored;

$$r_{ij}=\frac{a_{ij}}{\sum_{j=1}^{n}a_{ij}}$$ (1)

**Table 3:** Normalized matrix of Entropy.

	Agility	Cost	E-services	Financial innovation	Competitive environment	Risk optimization	Workflow management	Managerial decisions	Credibility	Bank Profitability	Robustness
A_1_	0.057	0.131	0.068	0.184	0.178	0.089	0.171	0.196	0.045	0.128	0.074
A_2_	0.109	0.071	0.193	0.053	0.100	0.204	0.104	0.152	0.089	0.163	0.170
A_3_	0.174	0.161	0.114	0.105	0.186	0.067	0.125	0.054	0.156	0.051	0.147
A_4_	0.082	0.190	0.091	0.224	0.040	0.133	0.185	0.109	0.067	0.222	0.085
A_5_	0.131	0.060	0.159	0.079	0.134	0.044	0.200	0.096	0.201	0.103	0.168
A_6_	0.164	0.095	0.134	0.132	0.140	0.196	0.050	0.147	0.111	0.077	0.204
A_7_	0.087	0.173	0.182	0.066	0.080	0.111	0.090	0.065	0.197	0.146	0.043
A_8_	0.196	0.119	0.059	0.158	0.142	0.156	0.075	0.182	0.134	0.110	0.109

*Step 3*. *Entropy and diversification outputs*. The entropy and diversification values are determined by the entropy strategy’s Formulae (2 and 3). Calculating diversification rates requires entropy ratios, which were determined before the diversification outputs. The sum of diversification values is also calculated. The required values are effectively determined in this step. Table 4 includes these findings in the proper places;

$$entropy\;(e)=-h\left(\sum_{i=1}^{m}r_{ij}*ln(r_{ij})\right)$$ (2)

herej=1,2,……n. As we know h = 1/ln (m), where m indicates a set of alternatives.

     So, h = 1/ln (8), hence, h = 1/ 2.0794

          h = 0.4809 and–h = - 0.4809

d=1−e (3)

**Table 4:** Entropy and diversification scores.

	Agility	Cost	E-services	Financial innovation	Competitive environment	Risk optimization	Workflow management	Managerial decisions	Credibility	Bank Profitability	Robustness	∑ d
A_1_	-0.163	-0.266	-0.183	-0.312	-0.307	-0.215	-0.302	-0.319	-0.139	-0.263	-0.193	
A_2_	-0.242	-0.189	-0.318	-0.155	-0.23	-0.325	-0.235	-0.287	-0.216	-0.296	-0.301	
A_3_	-0.305	-0.294	-0.247	-0.237	-0.313	-0.181	-0.26	-0.158	-0.29	-0.152	-0.282	
A_4_	-0.205	-0.316	-0.218	-0.335	-0.129	-0.269	-0.312	-0.241	-0.181	-0.334	-0.21	
A_5_	-0.266	-0.168	-0.292	-0.2	-0.269	-0.138	-0.322	-0.224	-0.322	-0.234	-0.3	
A_6_	-0.296	-0.224	-0.269	-0.267	-0.275	-0.319	-0.15	-0.282	-0.245	-0.197	-0.324	
A_7_	-0.213	-0.303	-0.31	-0.179	-0.202	-0.244	-0.217	-0.178	-0.32	-0.281	-0.134	
A_8_	-0.32	-0.253	-0.167	-0.291	-0.277	-0.289	-0.194	-0.31	-0.269	-0.243	-0.241	
$\sum_{i=1}^{m}r_{ij}*ln(r_{ij})$	-2.008	-2.013	-2.005	-1.976	-2.003	-1.98	-1.992	-1.999	-1.981	-2	-1.986	
**E**	0.966	0.968	0.964	0.95	0.963	0.952	0.958	0.961	0.953	0.962	0.955	
**d = 1—e**	0.034	0.032	0.036	0.05	0.037	0.048	0.042	0.039	0.047	0.038	0.045	**0.447**

*Step 4*. *Weights of criteria*. Algorithm (4) is a way to generate the weights for the criteria by dividing the diversification ratio of every column of Table 4 by their aggregate. The identified weights of each criterion are as displayed in Table 5.


$$Weight\;vector\;(W)=\frac{d}{\sum d}$$ (4)

**Table 5:** Criteria weights.

Criteria	Weights	Weights in Percent (%)
Agility	0.076	7.633
Cost	0.072	7.159
E-services	0.080	8.022
Financial innovation	0.111	11.086
Competitive environment	0.082	8.228
Risk optimization	0.107	10.694
Workflow management	0.094	9.398
Managerial decisions	0.086	8.639
Credibility	0.106	10.552
Bank profitability	0.085	8.510
Robustness	0.101	10.079

**Fig 5:**
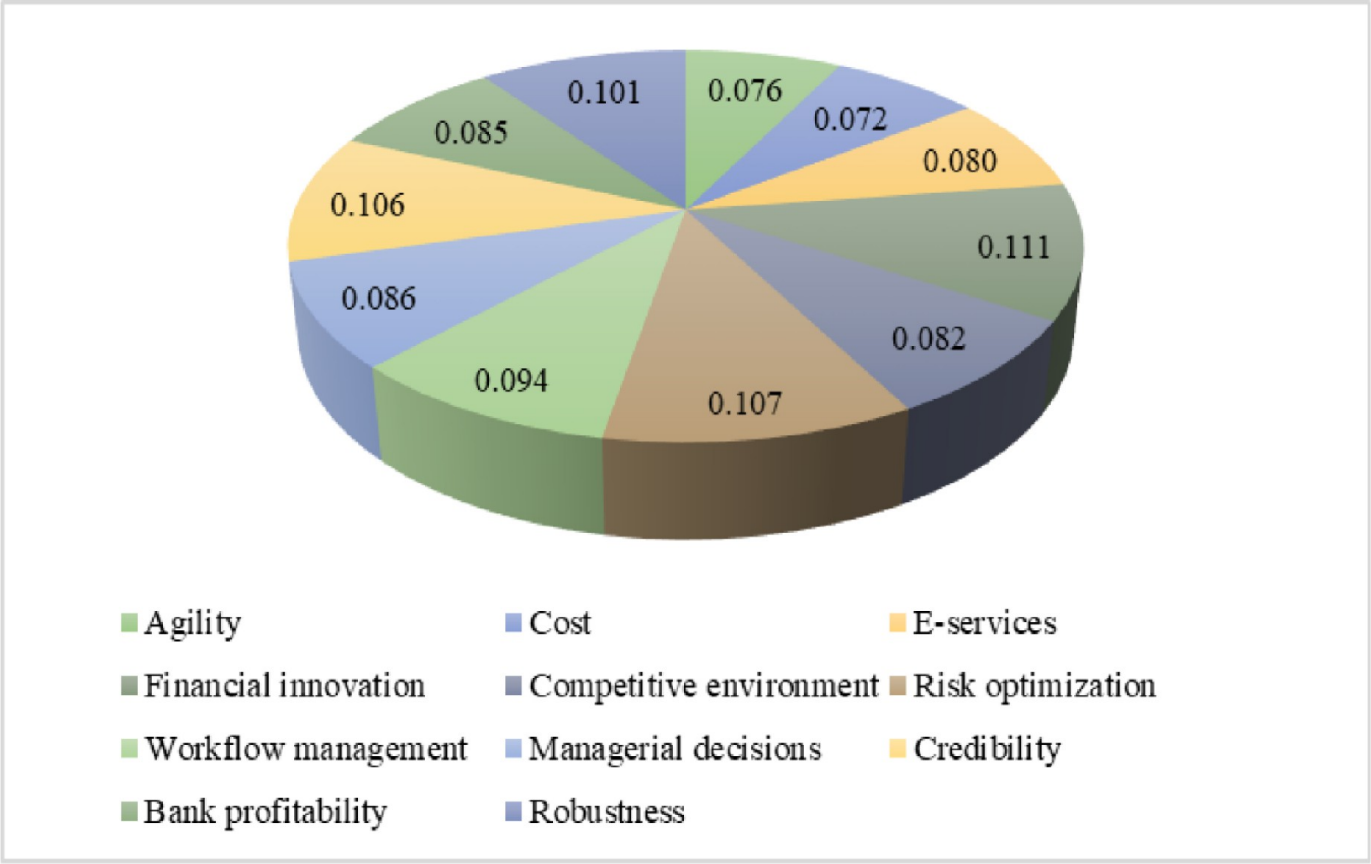
Criteria weights.

The weightage of every criterion chosen in this procedure is as depicted in Fig 5.

### TOPSIS technique

**Fig 6:**
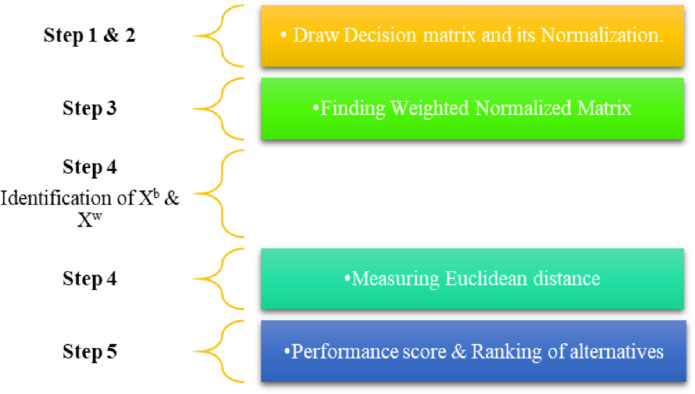
Steps involved in TOPSIS.

The TOPSIS approach is a prominent MCDM technique that meticulously evaluates options based on how close they are to an ideal solution. The initial stage in this algorithmic paradigm is to construct a decision matrix. Obtaining a variety of options together and assessing them based on certain criteria is necessary for this. Normalization of this matrix is important because it makes it easier to assess each option objectively and allows for comparison. The ideal or best and non-ideal or worst solutions for every criterion are found after the normalization procedure. Non-ideal solutions reflect the least or worst outcomes, whereas ideal solutions reflect the most beneficial or best outcomes. Taking every option’s distance from both the ideal and non-ideal solutions is the next step. This computation is considered necessary to determine the proximity of each choice to the ideal state. The spatial separation between each alternative and the optimal solution is subsequently determined. The alternatives are then assessed in relation to this proximity, with the alternative exhibiting a higher value being regarded as more appropriate. TOPSIS serves as a valuable approach for appraising diverse FinTech innovations or solutions within the banking sector, predicated on their efficacy in enhancing operational efficiency or customer satisfaction. The steps involved in this operation are as described in Fig 6.

#### Numerical work of TOPSIS

In this research paper, the evaluation and influential role of FinTech adoption, HR competency potential, service innovation, and firm growth in the banking sectors were completed by using Entropy-based TOPSIS processes. The TOPSIS mechanism is applied for the evaluation and ranking of different kinds of alternatives, which are selected for the assessment procedure. The alternatives (A_1_ to A_8_) that are evaluated and compared are arranged sequentially to make the selection among numerous options easy. It consists of different kinds of easily implemented equations that help us in the efficient evaluation and ranking of different alternatives. The strategy utilized in this research is related to eight potential results and eleven crucial elements that have significant impacts on the growth of banking sectors. The data obtained can be utilized to assess and put forth the eight solutions, sometimes referred to as Alternatives (A_1_ to A_8_). The single cost criterion is non-beneficial in contrast to all the other factors. The model incorporates the outcomes of a detailed investigation. Additionally, both TOPSIS-Entropy approaches employ the same decision matrix such as the Table 2 has also been utilized for the calculation of TOPSIS strategy. The step-by-step mechanism of this process are as illustrated below;

*Step 1*. *Normalized matrix*. The normalized scoring system was computed by utilizing the following technique (5), as well as the matrix is listed in Table 6. This is performed to reduce subjectivity and remove errors from the original matrix. The criterion weights are also displayed in the same table.


$$\alpha_{ij}=\frac{a_{ij}}{\sqrt{\sum_{i=1}^{n}(a_{ij}{)}^{2}}}$$ (5)

**Table 6:** Normalized matrix of TOPSIS.

Weights	0.076	0.072	0.080	0.111	0.082	0.107	0.094	0.086	0.106	0.085	0.101
**Criteria** **Alternatives**	Agility	Cost	E-services	Financial innovation	Competitive environment	Risk optimization	Workflow management	Managerial decisions	Credibility	Bank Profitability	Robustness
A_1_	0.150	0.349	0.180	0.475	0.472	0.231	0.448	0.515	0.116	0.337	0.194
A_2_	0.289	0.190	0.511	0.136	0.265	0.530	0.271	0.401	0.232	0.428	0.444
A_3_	0.463	0.428	0.301	0.271	0.494	0.173	0.327	0.143	0.405	0.135	0.383
A_4_	0.217	0.507	0.240	0.576	0.106	0.346	0.484	0.286	0.174	0.583	0.222
A_5_	0.347	0.159	0.421	0.203	0.356	0.115	0.523	0.252	0.521	0.270	0.439
A_6_	0.434	0.254	0.355	0.339	0.372	0.507	0.131	0.387	0.289	0.202	0.533
A_7_	0.231	0.460	0.481	0.170	0.212	0.288	0.235	0.172	0.512	0.384	0.111
A_8_	0.520	0.317	0.156	0.407	0.377	0.403	0.196	0.478	0.347	0.290	0.283

*Step 2*. *Weighted normalized matrix*. The scores for the Entropy-acquired parameters are multiplied by every parameter in the normalized selection matrix to form the weighted normalized matrix by applying Eq (6), as illustrated in Table 7.


$$=\left[\begin{array}{ccc} W_{11} & \cdots & W_{1n}\\ \vdots & \ddots & \vdots \\ W_{m1} & \cdots & W_{mn} \end{array}\right]\;Where\;X_{ij}=\alpha_{ij}*W_{j}$$ (6)

**Table 7:** Weighted normalized matrix.

	Agility	Cost	E-services	Financial innovation	Competitive environment	Risk optimization	Workflow management	Managerial decisions	Credibility	Bank Profitability	Robustness
A_1_	0.011	0.025	0.014	0.053	0.039	0.025	0.042	0.045	0.012	0.029	0.020
A_2_	0.022	0.014	0.041	0.015	0.022	0.057	0.026	0.035	0.024	0.036	0.045
A_3_	0.035	0.031	0.024	0.030	0.041	0.018	0.031	0.012	0.043	0.011	0.039
A_4_	0.017	0.036	0.019	0.064	0.009	0.037	0.045	0.025	0.018	0.050	0.022
A_5_	0.026	0.011	0.034	0.023	0.029	0.012	0.049	0.022	0.055	0.023	0.044
A_6_	0.033	0.018	0.028	0.038	0.031	0.054	0.012	0.033	0.031	0.017	0.054
A_7_	0.018	0.033	0.039	0.019	0.017	0.031	0.022	0.015	0.054	0.033	0.011
A_8_	0.040	0.023	0.013	0.045	0.031	0.043	0.018	0.041	0.037	0.025	0.029

*Step 3*. *Ideal best and worst scores*. To get the best and worst measures, apply Eqs (7) and (8). For non-beneficial criteria, the minimum values will be best and the maximum value will be the worst one.

$$X_{j}^{b}={max}_{i=1}^{n}\;X_{ij}$$ (7)

$$X_{j}^{w}={min}_{i=1}^{n}\;X_{ij}$$ (8)

Table 8 displays the findings attained from Eqs (7) and (8). Here, the cost criterion is non-beneficial that’s why shows the minimum value is in the best row while the higher is in the worst row.

**Table 8:** Ideal best and worst values.

X^b^	0.040	0.011	0.041	0.064	0.041	0.057	0.049	0.045	0.055	0.050	0.054
X^w^	0.011	0.036	0.013	0.015	0.009	0.012	0.012	0.012	0.012	0.011	0.011

*Step 4*. *Computing the separation*. The following formula serves to gauge ideal and nonideal measures by utilizing Eqs (9 and 10). The values obtained from these equations are as listed in Table 9.


$$S^{+}=\sqrt{\sum_{J=1}^{n}(X_{ij}-X_{j}^{b}{)}^{2}}$$ (9)

$$S^{-}=\sqrt{\sum_{J=1}^{n}(X_{ij}-X_{j}^{w}{)}^{2}}$$ (10)

**Table 9:** Separation measures.

Alternatives	$\sum_{J=1}^{n}(X_{ij}-X_{j}^{b}{)}^{2}$	S^+^	$\sum_{J=1}^{n}(X_{ij}-X_{j}^{w}{)}^{2}$	S^-^	S^+^ + S^-^
A_1_	0.0063	0.080	0.0049	0.070	0.149
A_2_	0.0049	0.070	0.0061	0.078	0.148
A_3_	0.0065	0.081	0.0040	0.064	0.144
A_4_	0.0058	0.076	0.0059	0.077	0.153
A_5_	0.0054	0.073	0.0063	0.079	0.152
A_6_	0.0042	0.065	0.0064	0.080	0.145
A_7_	0.0079	0.089	0.0035	0.059	0.148
A_8_	0.0041	0.064	0.0053	0.073	0.137

*Step 5*. *Performance score and ranking*. A grade is assigned to each alternative (A_1_ to A_8_) based on the progress made in the stage before. The values of the selected alternatives are determined by applying the Formula (11). Finally, we arranged the chosen alternatives in chronological order. The results obtained from the following equation and the alternatives’ ranking are as displayed in Table 10.


$$Performance\;score\;(P_{i})=\frac{{S_{i}}^{-}}{\left({S_{i}}^{+}+{S_{i}}^{-}\right)}$$ (11)

**Table 10:** Scores and grading of alternatives.

Alternatives	Score	Percentage	Rankings
A_1_	0.468	46.80	6
A_2_	0.528	52.83	3
A_3_	0.441	44.11	7
A_4_	0.504	50.36	5
A_5_	0.520	51.98	4
A_6_	0.553	55.33	1
A_7_	0.398	39.83	8
A_8_	0.531	53.11	2

**Fig 7:**
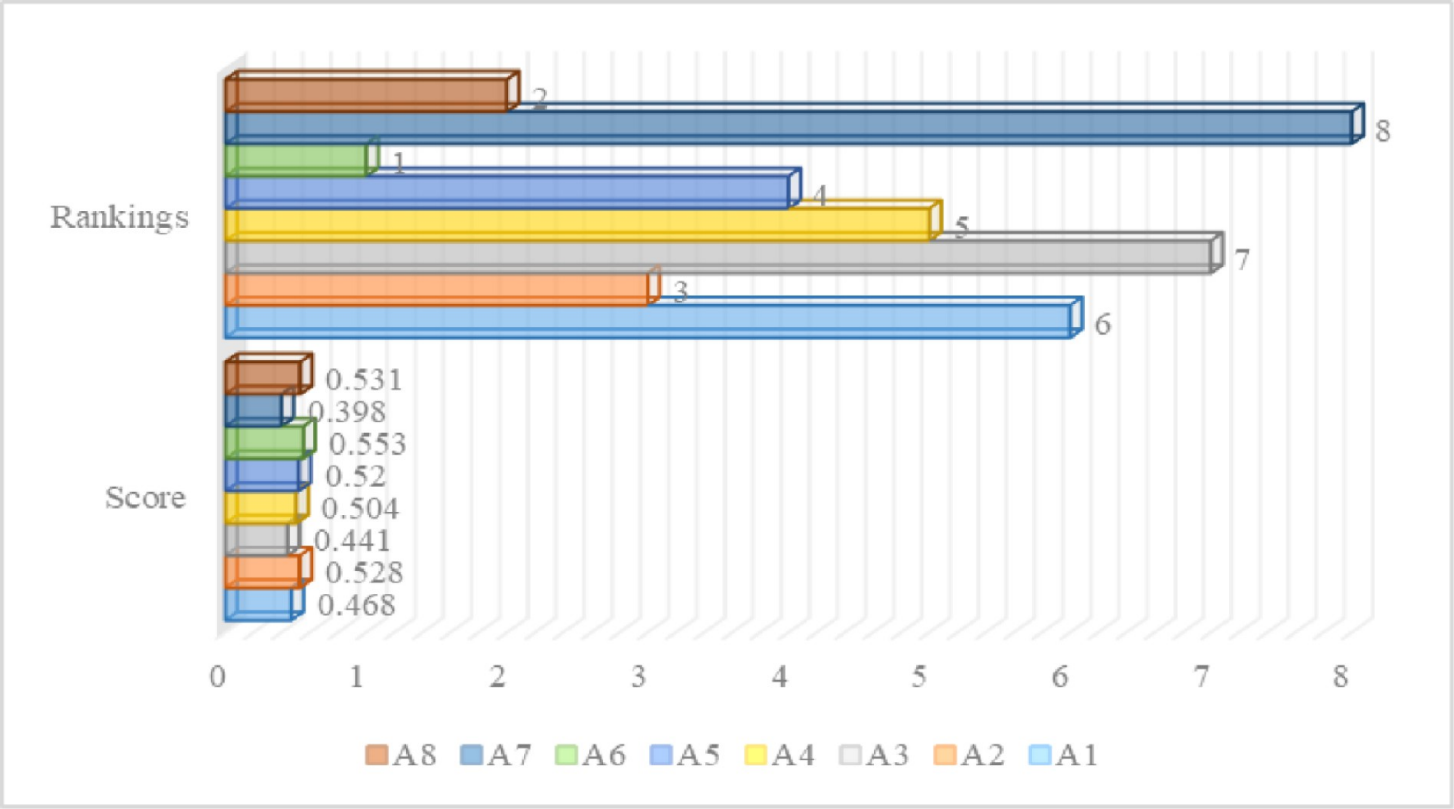
Score and ranking of alternatives.

The identified scores along with the rankings of the chosen alternatives are as described in Fig 7.

## Results

The banking sectors have widely advanced their component and increased their revenue due to the rapid development and adoption of digital technologies including FinTech and service innovation. These evolving components, which include HR competency potential, firm growth, service innovation, and FinTech adoption, have played a potential role and brought numerous positive advances in the financial and banking system. Due to different innovations in the banking sector, it becomes tough and challenging for researchers to evaluate the HR competency potential, firm growth, service innovation, and FinTech adoption in the banking sector. In this sense, the selection of a suitable alternative becomes necessary in the banking system. The hybrid MCDM-based approaches are used in this research to solve the evaluation and selection and make an efficient decision. For this purpose, previous publications related to this topic are thoroughly assessed and significant components of the banking sector are extracted for the selection and evaluation of a suitable alternative that has played a significant and influential role in the banking sector. A speedier and more accurate performance appraisal system has come about as the outcome of the adoption of integrated MCDM methodologies. A group of chosen candidates is eventually arranged consecutively once the weight of the parameter in this assessment has been defined. A hybrid MCDM framework integrates relevant multifaceted characteristics, such as cost, financial innovation, agility, and managerial decisions, to promote the study’s objectives and these factors providing a base for the rankings. This technique ensures an in-depth analysis capturing every aspect of each option’s impact on the combination of FinTech and human resource competencies.

**Fig 8:**
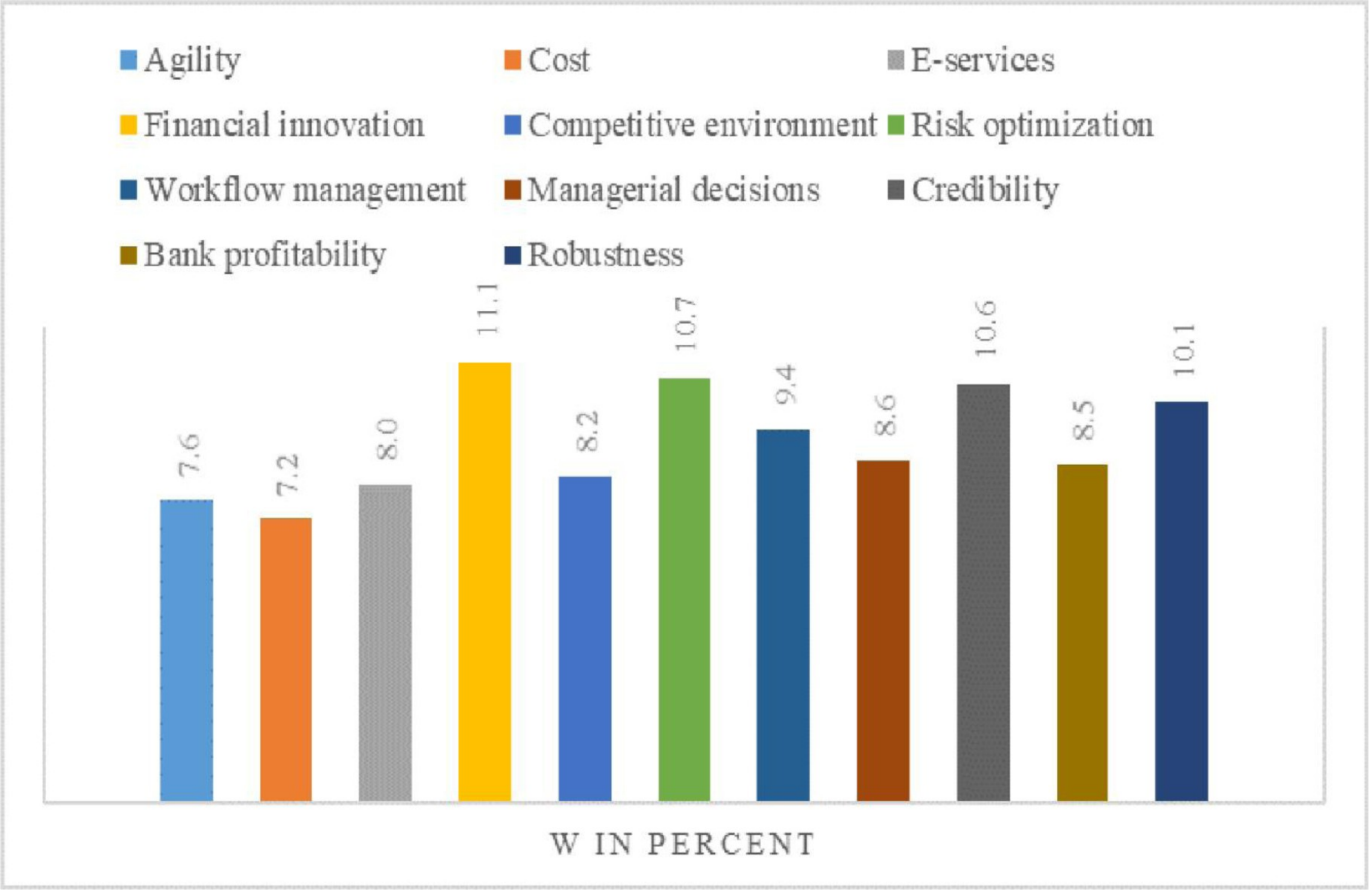
Criteria weights in percent.

The major effort of this evaluative study is to examine the FinTech adoption, service innovation, firm growth, and HR competency potential in banking. The ranking and selection of different kinds of alternatives chosen for evaluation are obtained by applying MCDM-based entropy and TOPSIS techniques. The EWM approach leverages the relative weights assigned to every criterion and the amount of data it communicates to methodically assess the importance of different factors. Consequently, the domains that have the most impact on financial technology integration and HR competencies are prioritized in the final rankings. The findings of the Entropy investigation show that the financial innovation criterion has a leading weight among all criteria with a maximum outcome of 11.1%. The remaining criteria along with their weights are as follows: risk optimization with a weight of 10.7%, credibility with a weight of 10.6%, robustness with a weight of 10.1, workflow management with a weight of 9.4%, managerial decisions with a weight of 8.6%, bank profitability with a weight of 8.5%, e-services with a weight of 8.0%, agility with a weight of 7.6%, and the cost with the lowest weight of 7.2%, as illustrated in Fig 8. The weight of each criterion indicates their importance and impact on the alternatives in the banking industry where the higher weight mean the greater importance while the lower weight mean the least importance in the banking sector. These values show the relative strength of the specified multifaceted characteristics, which helps in the ranking of alternatives to make a well-informed decision in the baking sector. These findings reveal that the financial innovation criterion has a higher influence on the performance and efficiency of alternatives.

**Fig 9:**
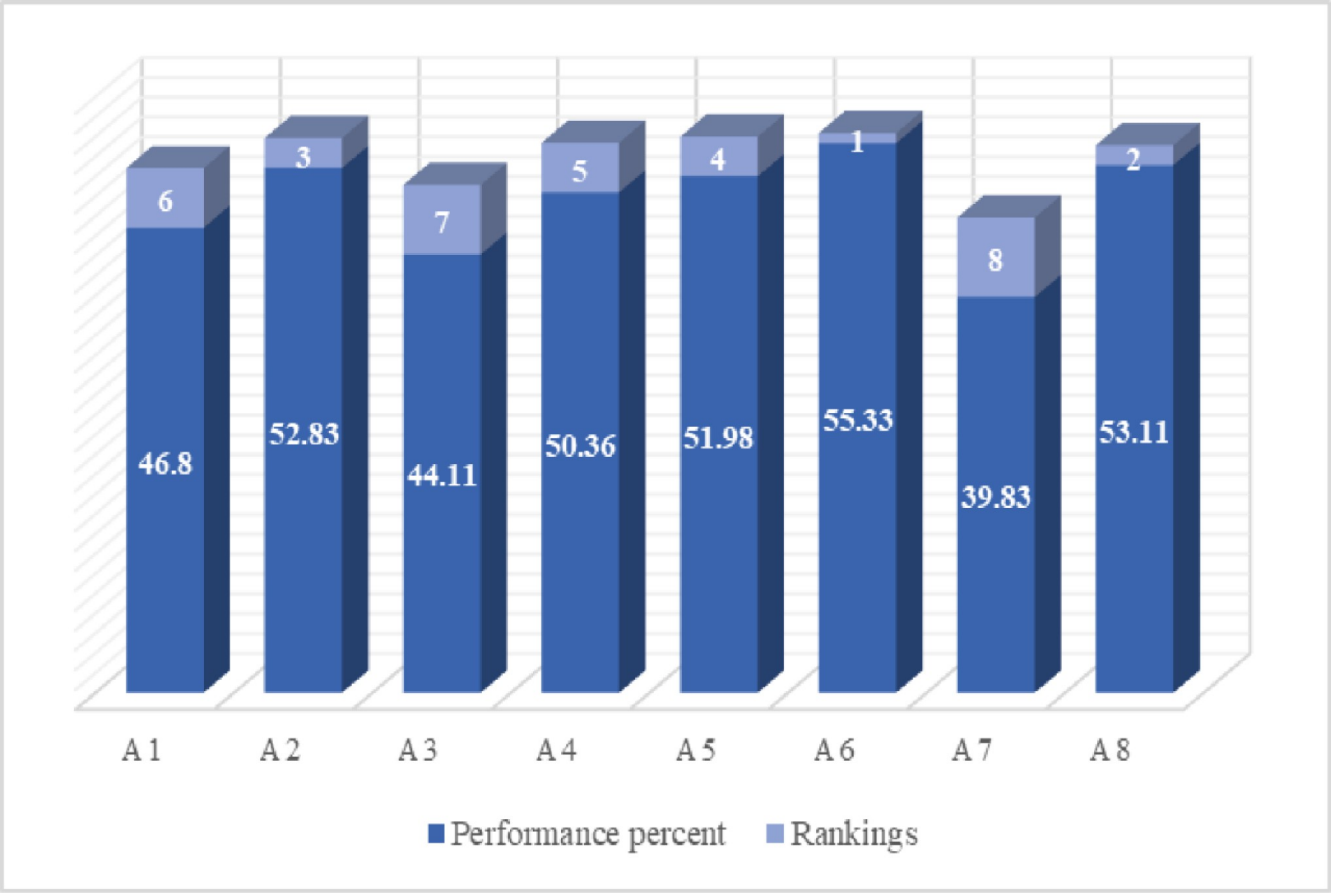
Alternatives score and ranking.

Meanwhile, the TOPSIS strategy, which analyzes options based on how far they deviate from an idealized solution, finds altenatives that are highly effective at promoting technological advancement and raising the efficiency of HR. The performance score and ranking of a set of different alternatives are obtained by using TOPSIS. This approach ranks a set of different alternatives based on their overall performance score in which the one having the highest score is placed at the top of the list by following the others. The result determined via the utilization of TOPSIS demonstrates that Alternative A_6_ is a leading one among numerous options with the highest outcome of 0.553, following the remaining options such as A_8_ placed 2^nd^ with a result of 0.531, A_2_ placed 3^rd^ with an outcome of 0.528, A_5_ placed 4^th^ with a value of 0.520, A_4_ placed at 5^th^ with a score of 0.504, A_1_ placed at 6^th^ with a value of 0.468, and A_3_ secure 7^th^ placed with an outcome of 0.441, whereas the alternative A_7_ has the worst one with the lowest outcome of 0.398 and placed at last in a list of alternatives as listed in Fig 9. The one having the highest rank can make significant contribution to the environmental efficiency of the banking industry by considering the specified multifaceted characteristics. It is found from the results that A_6_ is a top-performing alternative which means that it is performing better than others in the particular categories like firm growth, service innovation, FinTech adoption, and HR competency potential and further it has positive influence on the environmental efficiency of the banking system.

The evaluation findings indicate that the alternative A_6_ has played a potential role in the growth of the banking sector and become very beneficial for them in terms of saving operational costs, generating good profit, and time-saving. A_6_ is a top-performing alternative or banking institution among others renowned for its innovative methods and technological integration. Its agility is attributed to its flexible IT infrastructure, which enables prompt responses to changes in the market and customer demands. It uses AI and machine learning technologies to appraise customer data and create personalized financial goods. Its enhanced workflow management, which raises operational efficiency, is attributable to automation and digital technology. A_6_’s performance may be attributed to strategic investments in digital banking and fintech, which lower operational costs and attract a wider customer base. Its consumer engagement strategies, which include data analytics and customer relationship management technologies, strengthen client connections, encourage loyalty, and encourage higher usage of its financial products. The incorporated approaches provide strategic solutions for improving technology competency and HR performance in the banking industry. It reveals the interactions between FinTech and HR features, helping stakeholders understand the best options and factors contributing to their effectiveness. This comprehensive assessment aims to tackle major research goals by combining these methods. These findings and rankings with be extremely helpful for bankers and executives in the decision-making and evaluation of the relevant scenarios or issues in the upcoming years.

## Discussion

The results of the assessment of FinTech acceptability, the potential of HRC competencies, service innovation, and organizational growth in the banking industry have been compared with the body of existing research to resolve methodological and contextual imbalances. Previous work has employed several procedures, such as qualitative analyses, decision-making techniques, regression models, and case studies, which might have resulted in various interpretations of the same issue. This study uses a MCDM paradigm that combines the TOPSIS with entropy to establish a solid mathematical foundation for assessing and ranking options. The adoption of entropy in criteria importance analysis has become less common than in the past; instead, previous research efforts have mostly focused on more straightforward ranking or scoring systems. Due to this, the research utilizes hybrid MCDM analytical methodologies: TOPSIS and Entropy methods to analyze high-dimensional and non-linear correlations in banking sector data. It examines eleven attributes and eight possible choices, providing a comprehensive analysis of factors influencing FinTech adoption, HR competency potential, service innovation, and firm growth. Entropy is used for computing weighted criteria and TOPSIS for ordering alternatives, ensuring transparency and reproducibility. Entropy assesses the importance of each criterion, while Entropy manages data uncertainty and variability. TOPSIS ranks alternatives based on performance values, facilitating decision-making. The results show the effectiveness of integrated MCDM approaches in evaluating FinTech adoption, HR competency, service innovation, and firm growth in the banking sector. It is anticipated that this data-driven selection paradigm will offer valuable data about the variations in results, as previous studies might not have sufficiently tackled the complexities present in the banking industry.

However, the intersection of Fintech adoption, HR competency potential, service innovation, and firm growth and their applications in the banking sector is obtained by integrating MCDM-based approaches. An effective alternative among several options is determined that have played a competitive role in the banking sector and might be very helpful for banking systems to enhance their profit and reduce their operational costs. The literature review portion makes us realize that there are less number of publications available on this theme and very rare work found on the MCDM-based entropy-TOPSIS techniques. Earlier, it was a very big issue and strain for bankers and scholars to make a better decision among several options and efficiently evaluate different kinds of technologies. In this regard, our proposed effort solves these MCDM-related issues and will help bankers in their hard critical situations where making a decision is complex. The importance and ranking of each alternative chosen in this research are accomplished by using a hybrid MCDM paradigm. The results of this investigation will be used as assistance for bankers and executives when an evaluation-relevant issue arises and when making a better decision is challenging. The current effort proposed by us will provide a straightforward path to bankers and support them during the appraisal and decision scenarios on the subject related to the intersection of organization growth, service innovation, FinTech adoption, and HR competency potential in the banking sector.

The results of this investigation vary from previous research because of different methodological frameworks, contextual factors, and technological developments in the banking sector. The MCDM paradigm suggests that banking organizations reevaluate their development strategies for digital transformation, emphasizing the necessity of merging FinTech and HR potential. Following this assessment, bankers and executives were more adept at comprehending the paper’s ongoing efforts to support FinTech adoption, business development, service innovation, and HR competence potential in the banking industry and incorporate a reasonable strategy for the development of the banking system. The success of the banking industry will be improved by investors choosing and implementing a superior option that may increase profits and lower operating costs. For the decision-making and analysis of the potential for FinTech acceptability, company development, service innovation, and HR competency in the banking industry, the present approach used MCDM-based techniques and gave comprehensive evidence and precise findings. The investigation’s significant dimension is the intentional sequencing of the different kinds of alternatives’ involvement to ensure a more satisfying conclusion using entropy-TOPSIS methodologies. Strengthening these concepts in the dynamic industry will require more research.

## Conclusions

The primary intent of this paper is to ensure the availability of the analysis methods that assist bankers and investors throughout the assessment of FinTech adoption, firm growth, the potential for HR competency, and service innovation within the banking sector disciplines. The banking industry is leveraging FinTech adoption, business expansion, HR competency potential, and service innovation to enhance growth and improve efficiency. These technologies have enhanced competitiveness, reduced operational costs, and reduced operational costs. The adoption of these technologies streamlines activities, maintains accuracy, increases profits, and saves expenditures, contributing to the industry’s overall success. The rapid implementation of FinTech in the banking sector has made it challenging for scholars and bankers to evaluate its adoption, HR competency potential, and service innovation. To address this, an integrated MCDM technique is presented, which efficiently evaluates and determines the best possible alternative among numerous options. After the extraction of some significant features from the existing studies through an in-depth review, we then applied entropy and TOPSIS techniques to determine the criterion importance and rank the alternatives for better decision-making in the banking sector. The weightage of criteria has been attained by using entropy, whereas the ranking of a set of different alternatives has been attained by using TOPSIS. The results find the lucrative and premium alternative that has made a significant contribution to the upbeat expansion of the banking industry. After a thorough assessment of Fintech and service innovation in the banking sector, the findings reveal that alternative A_6_ has played a very impressive role in the development of the banking sector and has a higher potential to increase the revenue of the financial systems and bring innovative changes in this sector. On the other hand, alternative A_7_ is known as the worst alternative because of its lowest value and has a negative impact on the growth of the banking sector. MCDM techniques reduced multi-criteria issues’ complexity and eliminated computational errors, enhancing decision-making and enabling the selection of the best alternative from a variety of choices, thus empowering analyzers. Numerous significant findings arise from the analysis like the entropy and TOPSIS techniques have been used to prioritize and assess alternatives, enabling smart decision-making and efficient resource allocation. The research further demonstrates the significance of HR competency in overseeing technological advancements and promoting expansion inside financial establishments as well as revealing the need for sustainable banking mechanisms that maintain a balance between social and environmental obligations and economic advancement. One useful tool and theoretical foundation for promoting innovation and progress in the banking sector is the utilization of a hybrid MCDM paradigm. It may be applied to a wide range of tasks, such as assessing technology solutions based on predetermined standards, adopting FinTech, improving HR capabilities, service innovation, and firm growth. This systematic strategy enhances client satisfaction and retention metrics while streamlining the financing process. One relevant example is a global financial institution that implemented this process to assess potential collaborations with FinTech startups based on their technological competence and alignment with the bank’s strategic goals. The unique feature of this study is how th proposed MCDM strategies are used to sequentially arrange a group of competing options in order to arrive at a more favorable decision. This evaluative effort will be beneficial in critical situations or scenarios where the decision is hard to make and deal with tough situations where the selection among numerous options is not easy. The research suggests further investigation into the integration of developing technologies in the banking industry, aiming to enhance online presence and adapt to financial sector changes. The use of multi-criteria methodologies effectively addresses every dilemma in the future that can come up in the evaluation and selection process of the intersection of these cutting-edge technologies in the banking industry.

This research has some limitations, including the potential for not all factors to be covered by the specified standards as well as the dynamic nature of the financial technology industry necessitates periodic reevaluations to maintain the reliability of the weights and standards. Further research could expand the dataset to include diverse institutions from different markets and geographical areas and also focus on developing adaptable frameworks to keep up with changing consumer behavior and technology. Future research should investigate the application of the EWM-TOPSIS methodology in non-banking sectors like insurance or investment enterprises. Empirical case studies from actual corporations demonstrating effective incorporation of FinTech technologies within the banking sector could provide valuable insights and support the proposed strategies. The process’s robustness can be further increased by integrating revolutionary technologies like blockchain and AI into the assessment mechanism. Researchers investigating the impact of these technologies on service innovation and firm development may contribute to strengthening the MCDM paradigm.

## Supporting information

S1 File (XLSX)

S2 File (XLSX)

## References

[1] Liu C., et al., Distributed Neural Tensor Completion for Network Monitoring Data Recovery. Information Sciences, 2024: p. 120259.

[2] Habib Ullah Khan Muhammad Zain Malik, Nazir Shah, and Khan Faheem. "Utilizing bio metric system for enhancing cyber security in banking sector: A systematic analysis." IEEE Access (2023).

[3] Campanella F., et al., FinTech in the financial system: Towards a capital-intensive and high competence human capital reality? Journal of Business Research, 2023. 155: p. 113376.

[4] King T. and Previati D.A., FinTech Cultures and Organizational Changes in Financial Services Providers, in Disruptive Technology in Banking and Finance: An International Perspective on FinTech, King T, et al., Editors. 2021, Springer International Publishing: Cham. p. 195–219.

[5] Campanella, F., et al. Financial Technology: evidence in the European Banking System. in 2020 IEEE International Conference on Technology Management, Operations and Decisions (ICTMOD). 2020.

[6] Dubey V. and Walimbe R.S., Fintech 2022 Trends: The era of F-CUBE “Fast and Furious Fintech”. 2021.

[7] Akyuwen R., Nanere M., and Ratten V., Technology Entrepreneurship: Fintech Lending in Indonesia, in Entrepreneurial Innovation: Strategy and Competition Aspects, Ratten V, Editor. 2022, Springer Nature Singapore: Singapore. p. 151–176.

[8] Valeau P., et al., The mediating effects of professional and organizational commitment on the relationship between HRM practices and professional employees’ intention to stay. The International Journal of Human Resource Management, 2021. 32(8): p. 1828–1864.

[9] Gupta N. and Verma P.K.. A PLS-SEM Approach for Analysing Job Satisfaction and Human Resource Practices in Indian Banking Sector. in Proceedings of Second International Conference in Mechanical and Energy Technology. 2023. Singapore: Springer Nature Singapore.

[10] Naseer S., et al., How and when information proactiveness leads to operational firm performance in the banking sector of Pakistan? The roles of open innovation, creative cognitive style, and climate for innovation. International Journal of Information Management, 2021. 56: p. 102260.

[11] Mirza N., et al., The role of fintech in promoting green finance, and profitability: Evidence from the banking sector in the euro zone. Economic Analysis and Policy, 2023. 78: p. 33–40.

[12] Kitsios F., Giatsidis I., and Kamariotou M., Digital Transformation and Strategy in the Banking Sector: Evaluating the Acceptance Rate of E-Services. Journal of Open Innovation: Technology, Market, and Complexity, 2021. 7(3): p. 204.

[13] Singh J., et al., Big Data as a Service and Application for Indian Banking Sector. Procedia Computer Science, 2022. 215: p. 878–887.

[14] Khan Abdul Wahid, Zaib Shah, Khan Faheem, Tarimer Ilhan, Jung Taek Seo, and Jiho Shin. "Analyzing and evaluating critical cyber security challenges faced by vendor organizations in software development: SLR based approach." IEEE access 10 (2022): 65044–65054.

[15] Khan Shams Ullah, Abudul Wahid Khan Faheem Khan, Muhammad Adnan Khan, and Taeg Keun Whangbo. "Critical success factors of component-based software outsourcing development from vendors’ perspective: a systematic literature review." IEEE Access 10 (2021): 1650–1658.

[16] Khan Saad Ullah, Abdul Wahid Khan Faheem Khan, Khan Jawad, and Lee Youngmoon. "Factors influencing vendor organizations in the selection of DevOps for global software development: an exploratory study using a systematic literature review." Cognition, Technology & Work 25, no. 4 (2023): 411–426.

[17] Ahmad Jamshed, Ghazal Taher M., Abdul Wahid Khan Muhammad Adnan Khan, Inairat Mohammad, Sahawneh Nizar, and Khan Faheem. "Quality requirement change management’s challenges: an exploratory study using slr." IEEE Access 10 (2022): 127575–127588.

[18] Talpur A.B., Market power and concentration-performance analysis of the banking sector: A comparative study of Singapore and Pakistan. Social Sciences & Humanities Open, 2023. 7(1): p. 100383.

[19] Khan Faheem, Ahmad Shabir, Hüseyin Gürüler Gurcan Cetin, Whangbo Taegkeun, and Kim Cheong-Ghil. "An efficient and reliable algorithm for wireless sensor network." Sensors 21, no. 24 (2021): 8355. doi: 10.3390/s21248355 34960449 PMC8705826

[20] Fasano F. and Cappa F., How do banking fintech services affect SME debt? Journal of Economics and Business, 2022. 121: p. 106070.

[21] Marhraoui M.A. and El Manouar A., IT Innovation and Firm’s Sustainable Performance: The Mediating Role of Organizational Agility. Icime 2017, 2017: p. 150–156.

[22] Gabbasova B., Mavlyautdinov L., I. S, and Sabitova S. U, Digital Technologies in the System of Relations Competitiveness of Banks. Defin-2021, 2022.

[23] Kör B., The mediating effects of self-leadership on perceived entrepreneurial orientation and innovative work behavior in the banking sector. SpringerPlus, 2016. 5(1): p. 1829. doi: 10.1186/s40064-016-3556-8 27818867 PMC5074951

[24] Dwivedi P., Alabdooli J.I., and Dwivedi R., Role of FinTech Adoption for Competitiveness and Performance of the Bank: A Study of Banking Industry in UAE. International Journal of Global Business and Competitiveness, 2021. 16(2): p. 130–138.

[25] Thommandru A. and Chakka D.B., Recalibrating the Banking Sector with Blockchain Technology for Effective Anti-Money Laundering Compliances by Banks. Sustainable Futures, 2023. 5: p. 100107.

[26] Huang W., Wu Y., and Deng L., Does banking competition stimulate regional innovation? Evidence from China. Pacific-Basin Finance Journal, 2021. 70: p. 101674.

[27] Boikos S., et al., Financial reforms and innovation: A micro–macro perspective. Journal of International Money and Finance, 2023. 132: p. 102820.

[28] Thampy A. and Tiwary M.K., Local banking and manufacturing growth: Evidence from India. IIMB Management Review, 2021. 33(2): p. 95–104.

[29] Brodny J. and Tutak M., Analyzing the Level of Digitalization among the Enterprises of the European Union Member States and Their Impact on Economic Growth. Journal of Open Innovation: Technology, Market, and Complexity, 2022. 8(2): p. 70.

[30] Brodny J. and Tutak M., Assessing the level of innovativeness and digitalization of enterprises in the European Union States. Journal of Open Innovation: Technology, Market, and Complexity, 2024. 10(1): p. 100210.

[31] Marino-Romero J.A., Palos-Sánchez P.R., and Velica-Martín F., Evolution of digital transformation in SMEs management through a bibliometric analysis. Technological Forecasting and Social Change, 2024. 199: p. 123014.

[32] Brodny J. and Tutak M Digitalization of Small and Medium-Sized Enterprises and Economic Growth: Evidence for the EU-27 Countries. Journal of Open Innovation: Technology, Market, and Complexity, 2022. 8, DOI: doi: 10.3390/joitmc8020067

[33] Kljajić Borštnar M. and Pucihar A Multi-Attribute Assessment of Digital Maturity of SMEs. Electronics, 2021. 10, DOI: doi: 10.3390/electronics10080885

[34] Cao B., et al., Applying graph-based differential grouping for multiobjective large-scale optimization. Swarm and Evolutionary Computation, 2020. 53: p. 100626.

[35] Peng J.J., et al., Picture fuzzy decision-making theories and methodologies: a systematic review. International Journal of Systems Science, 2023. 54(13): p. 2663–2675.

[36] Khan H.U., et al., Multi-criteria decision-making methods for the evaluation of the social internet of things for the potential of defining human behaviors. Computers in Human Behavior, 2024. 157: p. 108230.

[37] Khan H.U., et al., Selection of a smart and secure education school system based on the internet of things using entropy and TOPSIS approaches. Computers in Human Behavior, 2024. 159: p. 108346.

