# Correction: The intersection of finTech adoption, HR competency potential, service innovation, and firm growth in the banking sectors using Entropy and TOPSIS

**DOI:** 10.1371/journal.pone.0320871

**Published:** 2025-03-20

**Authors:** Habib Ullah Khan, Muhammad Abbas, Shah Nazir, Faheem Khan, Yeon-kug Moon

The funding statement for this paper is incorrect. The correct funding statement is as follows: This work was supported by the Technology Innovation Program (RS-2024-00487049, Development and demonstration of complex emotion recognition on-device AI technology for in-vehicle driver emotional services), funded by the Ministry of Trade, Industry & Energy (MOTIE, Korea).
